# Conglobation in the Pill Bug, *Armadillidium vulgare*, as a Water Conservation Mechanism

**DOI:** 10.1673/031.008.4401

**Published:** 2008-05-28

**Authors:** Jacob T. Smigel, Allen G. Gibbs

**Affiliations:** ^1^ College of Osteopathic Medicine, Midwestern University, 19555 North 59th Ave., Glendale, Arizona USA 85308; ^2^ School of Life Sciences, University of Nevada, Las Vegas, Nevada USA 89154

**Keywords:** metabolic rate, terrestrial isopod, water loss, woodlouse

## Abstract

Water balance of the terrestrial isopod, *Armadillidium vulgare*, was investigated during conglobation (rolling-up behavior). Water loss and metabolic rates were measured at 18 ± 1°C in dry air using flow-through respirometry. Water-loss rates decreased 34.8% when specimens were in their conglobated form, while CO2 release decreased by 37.1%. Water loss was also measured gravimetrically at humidities ranging from 6 to 75 %RH. Conglobation was associated with a decrease in water-loss rates up to 53 %RH, but no significant differences were observed at higher humidities. Our findings suggest that conglobation behavior may help to conserve water, in addition to its demonstrated role in protection from predation.

## Introduction

Terrestrial isopods (Crustacea, Isopoda, Oniscidea) are successful colonizers of land that comprise over 1,000 species. Despite their diversity, they are relatively poorly adapted to land, and are confined to microhabitats where temperatures are moderate and damp surfaces are available (Spencer and Edney 1954; Hadley 1994). They seek shelter under fallen leaves and rotting wood where the water content of the soil is greater than 10% (Little 1983). Although they seek moisture, terrestrial isopods must also avoid overly moist conditions such as frequently flooded grasslands (Plum 2005; Hassall and Tuck 2007). They emerge primarily at night, or whenever the temperature drops and the relative humidity of the air increases, to forage for food.

Water loss to the environment is inevitable, as terrestrial isopods lack cuticular lipids and the elaborate spiracular apparatus of insects (Edney 1967; Hadley 1994). Most species rapidly lose body water by transpiration in all but nearly saturated air (Edney 1951; Spencer and Edney 1954; White and Zar 1968), although some can actively absorb water vapor above 90 %RH (Wright and Machin 1993a, b). Terrestrial isopods rely instead on chemoreceptors to locate suitable shelter and passively aggregate with members of their species (Warburg 1993). They obtain water from their food, and, when free water is available, active imbibe water through the mouth and anus (Cloudsley-Thompson 1977).

A major route for water loss is evaporation from the body surface, particularly the respiratory organs (Kuenen 1959). These “gill-like” organs, called pleopods, are located on the ventral abdominal segments (Edney 1951). To preserve their function in environments where the relative humidity is less than saturating, the outer surface of the pleopods must be kept moist (Cloudsley-Thompson 1977; Little 1983; Wright and Ting 2006). Evaporation from the pleopods is one of the most important physiological factors affecting the survival and distribution of isopods (Edney 1951, 1953, 1968; Cloudsley-Thompson 1969).

Though most terrestrial isopods seek similar microhabitats, they differ markedly in their ability to tolerate dry conditions. The families Oniscidae, Porcellionidae, and Armadillidiidae are found in progressively drier areas and show increasing morphological specialization (Cloudsley-Thompson 1977; Wright and Ting 2006). These families have more developed respiratory organs and better tolerate dry conditions than other isopods (Edney 1951; Kuenen 1959). For example, the marine littoral genus *Ligia* possesses simple plate-like pleopods, similar to those of aquatic isopods, while terrestrial genera such as *Porcellio* and *Armadillidium* have evolved invaginated lungs in the exopodites (Wright and Ting 2006). The invaginated lungs of *A. vulgare* form “pseudotracheae,” which are interpreted as an adaptation for respiration that allows *A. vulgare* to take up 94% of its normal oxygen requirement in dry air, when the integument is also dry (Cloudsley-Thompson 1977).

Among terrestrial isopods, the Armadillidiidae, commonly called “pill bugs,” are exceptional in that they are the only family capable of conglobation, or the ability to roll up into a ball. *Armadillidium vulgare* does this very well, drawing its antennae inside to form an uninterrupted sphere. This behavior can be triggered by strong vibrations or pressure and offers protection from predators such as shrews and spiders (Cloudsley-Thompson 1977). In the case of other predators, a paradox exists in that a great variety of animals are known to eat pill bugs under laboratory conditions, with little evidence of sustained predation in the wild (Sutton 1980).

In addition to protection from predation, conglobation also protects the more delicate ventral surface, allowing pill bugs to survive conditions that may be lethal to other species (White and Zar 1968). Evidence in this regard comes from Edney (1951), who found that both *A. nasatum* and *A. vulgare* spontaneously conglobate at 40 °C, with a resulting decrease in their rates of water loss. In these experiments, the only other temperatures investigated were above 40 °C, non-ecological conditions which ultimately proved lethal to the animals. Edney (1951) also measured water loss from *A. nastum* and *A. vulgare* that were held open at ≥ 40 °C, having been wrapped in wire mesh. The rate of water loss in this group was higher than expected, but Edney (1951) reported that the mesh may have damaged the cuticle, leading to an abnormally high rate of water loss.

In the work described here, initial field observations indicated that undisturbed *A. vulgare* tended to be found conglobated in dry soil, and unconglobated in visibly moist soil. This suggested that conglobation may also serve a water-conservation function. To test this hypothesis, we measured rates of water loss from unconglobated (free) and conglobated isopods. The data show that conglobation significantly reduced water loss from *A. vulgare*.

## Materials and Methods

### Animal collection and maintenance

Several hundred isopods were collected at a residence in January and February of 2006, in Las Vegas, Nevada. They were held in the dark at 18 °C in plastic bins containing 3 cm of moist soil from the collection site. The soil was frequently wetted in a consistent manner, and raw carrot slices were supplied as food. A subsample was selected at random and identified as *A. vulgare* by J. B. Knight (Entomologist, Nevada Department of Agriculture).

### Flow-through respirometry

Before experimentation, individuals were selected at random from the population and placed in individual wells of a microplate containing moist soil. They were no longer fed, but otherwise maintained in the same manner as the source population. Only inter-molt adults were chosen, as the cuticles of isopods are noticeably soft for several hours after molting (Warburg 1993). We observed that isopods that were actively molting (or had just finished molting) lost water so rapidly that they were seen to shrivel after only several minutes in the desiccating conditions of the respirometer. To avoid testing isopods that were molting, only those with dark, firmly attached cuticles were used in the experiments.

After 48 h of isolation, metabolic rates and water-loss rates were measured using flow-through respirometry. After being freed of attached soil, individuals were weighed on an analytical balance and placed in 5 ml glass-aluminum chambers, which were then placed in a Sable Systems (www.sablesys.com) TR-2 respirometer maintained at 18 ± 1°C. Rates of carbon dioxide release and water loss were measured with a Li-Cor (www.licor.com) LI-6262 infrared CO2 and water vapor sensor. Air was scrubbed of CO2 upstream of the gas analyzer using a commercial sodium hydroxide absorbent (Ascarite™, Thomas Scientific, www.thomassci.com/index.jsp), and of water vapor using anhydrous calcium sulfate. Air flow was maintained at 100 ml/min for the 30 min measurement period. Data were collected and analyzed using Datacan V software (Sable Systems). Water-loss rates were determined for 29 individuals, but because of a technical problem, metabolic rates were determined for only 20 animals.

**Figure 1.**
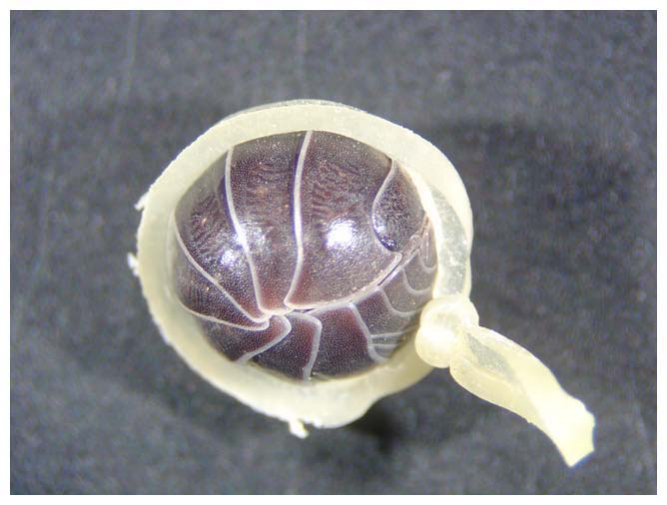
An isopod subjected to forced conglobation by a dental rubber band.

*Armadillidium vulgare* has been shown to survive loss of 25% of its body weight if given the opportunity to take up water again quickly (Kuenen 1959). With this in mind, individuals were immediately returned to their moist wells to recover from desiccation stress imposed during the respiration measurements. We used the same individuals for respirometry under conglobated and free conditions, with the order of measurement randomized among isopods. Conglobation was achieved by wrapping specimens in soft 8 mm orthodontic rubber bands (GAC International, Canada) that were knotted centrally to reduce their diameter (Figure 1). Two respiratory measurements were performed, with a 48 hr interval between them. This period between desiccation trials proved sufficient, as only three isopods died between trials, while those that lived increased their body weight by an average of 3.7%. Edney (1951) also noted that two species of *Armadillidium* frequently gained back more weight in saturating humidity than they had lost during desiccation.

### Water loss at known humidity

In a second experiment, isopods were randomly selected and isolated under the same conditions used for flowthrough respirometry. Isopods were held in moist conditions at 18 ± 1°C for 48 h, then each isopod was exposed, in both the free and conglobated state, to a relative humidity between 6 and 75 %RH for 3 h. Humidity was regulated in sealed glass vials containing 15 ml of a saturated salt solution (Winston and Bates 1960). Five saturated salt solutions, of varying humidity, were made from reagent-grade chemicals (Table 1). The relative humidity of each saturated solution was calculated by the formula RH = A^(B/T)^, where RH is the percent relative humidity (generally accurate to ± 2%), T is the temperature in Kelvin, and A and B are constants specific to each salt (Wexler 1992). Each vial was equipped with a centrally located foam plug which separated the isopod from the salt solution.

**Table 1.**
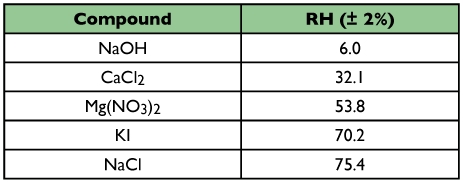
Percent relative humidity produced at 18 ± 1°C when an excess of a water-soluble salt is contained within an enclosed space (Wexler 1992).

Each isopod was weighed, before and after desiccation, on an analytical balance with a precision of 1 µg, to measure changes in body mass during exposure. Defecation was noted on only three occasions, and metabolic mass losses have been shown to be negligible (Wright and Machin 1993a), so the differences in mass were used to estimate non-excretory whole-body water loss. Isopods were placed in individual humidity vials to prevent aggregation, which could reduce the rate of water loss from one or more individuals (Allee 1926; Yoder and Grojean 1997; Yoder and Smith 1997).

Measurements in the free state were performed first. The isopods were then allowed to recover for 48 h before the procedure was repeated with all individuals in a conglobated state, which was achieved by the application of soft orthodontic rubber bands. Initial experiments revealed that the additional setup time and handling stress, inherent in the application of rubber bands, significantly affected total water loss in the conglobated experimental group. Isopods that were forced to conglobate expended more energy by struggling during the application of the rubber bands and spent more time outside of their saturated wells than the group that was allowed to remain free. The additional mass loss during handling would therefore tend to overestimate water loss from the conglobated animals. To correct for this, both groups of isopods were treated to the same handling conditions. A “sham-conglobation” was performed for the unconglobated treatment, in which all isopods were put through the extended exposure period and handling stress of forced conglobation with orthodontic rubber bands. These isopods were released from their rubber bands just as they were placed into their respective vials and became our free test group. Statistical analyses were performed using Statistica 7 (StatSoft, Inc., www.statsoft.com).

## Results

### Water-loss rates measured using flow-through respirometry

**Figure 2.**
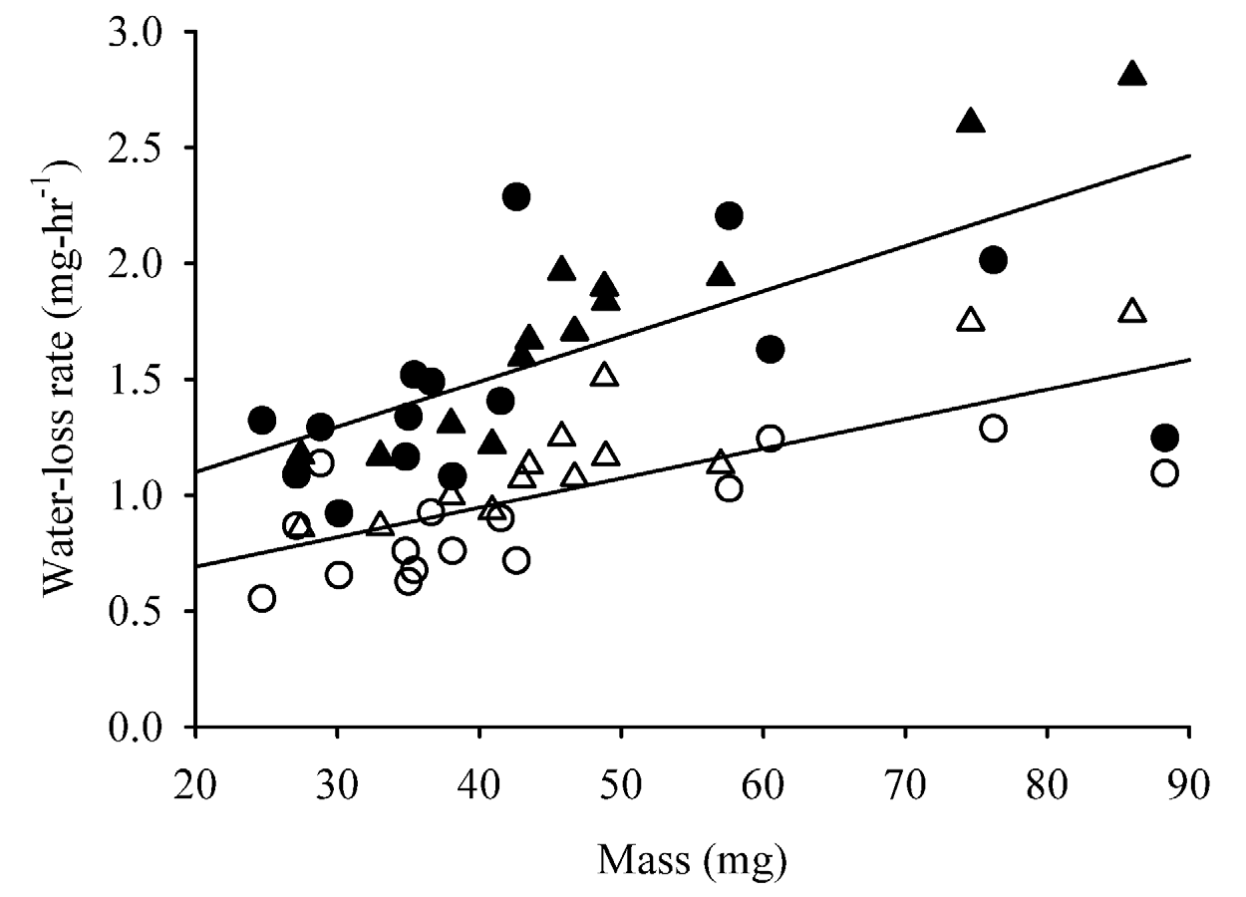
Rates of water loss from male and female *A.* *vulgare* in free and conglobated states. Symbols: Closed, free; open, conglobated; circles, females; triangles, males.

For every individual studied, water-loss rates were lower in the conglobated than the free state (Figure 2; n = 29, sign test P < 10^-6^). The average reduction in water loss was 34.8% (±2.5% SE, n = 29). Because water-loss rates from some individuals were measured first in the conglobated state, and others first in the free state, we performed initial analyses of variance for the free and conglobated states, separately, with measurement order and sex as main factors and mass as a covariate. These indicated that measurement order did not affect water-loss rate in either the free or conglobated state (F_1, 21_ < 0.8; P > 0.4 for both ANOVAs). A repeated-measures ANCOVA with sex and measurement order as factors and mass as a covariate also indicated that the order of measurement did not affect water-loss rates (Table 2). Not surprisingly, larger individuals lost water more rapidly than smaller ones. A significant effect of sex was found, with males tending to lose water faster, for their size, than females.

**Table 2.**
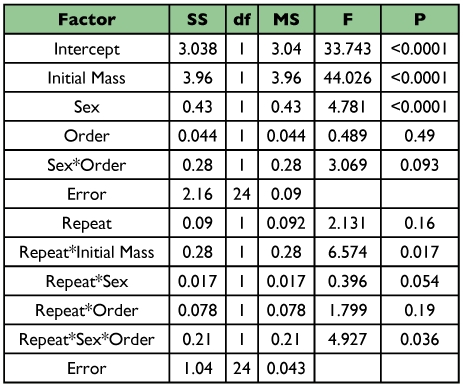
Repeated measures ANCOVA for water-loss rates from *A. vulgare*. Order refers to the order in which respirometry measurements were performed.

### Metabolic Rates

**Figure 3.**
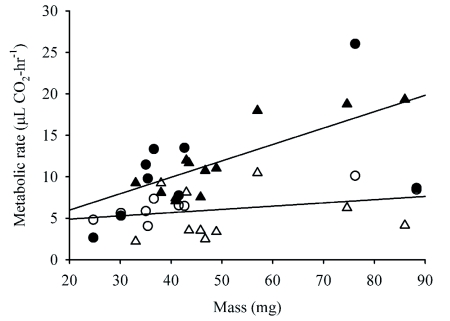
Rates of CO_2_ release in male and female *A. vulgare* in free and conglobated states. Symbols: Closed, free; open, conglobated; circles, females; triangles, males.

Metabolic rates were lower in the conglobated state than in the free state for 16 of 20 individuals studied (Figure 3; sign test, P < 0.02). The average decrease in CO_2_ release was 37.1% (±9.1% SE) in conglobated isopods. Initial analyses suggested that measurement order affected metabolic rates. This was confirmed by a repeatedmeasures ANCOVA (Table 3). However, inspection of the data revealed that this effect was dominated by a group of males with very low metabolic rates in the conglobated state (∼30 % of the free state). All of these had first been assayed in the free state, thereby generating the statistically significant repeat^*^sex^*^measurement order effect in Table 3. Water-loss measurements from these individuals did not exhibit a similar pattern, nor were respirometry measurements for these individuals performed on the same day, so the cause of this difference is unclear. In other animals (females and males assayed first while conglobated), CO_2_ release was ∼20 % lower in the conglobated state.

**Table 3.**
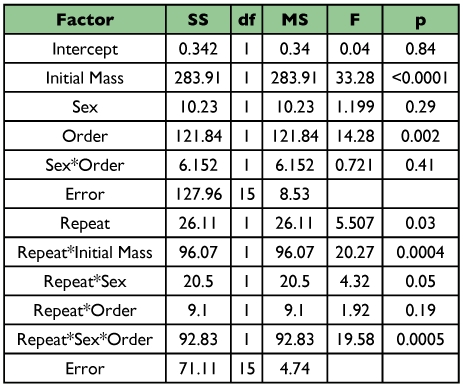
Repeated measures ANCOVA for CO_2_ release from *A. vulgare*. Order refers to the order in which respirometry measurements were performed.

### Water loss at sub-saturating humidity

**Figure 4.**
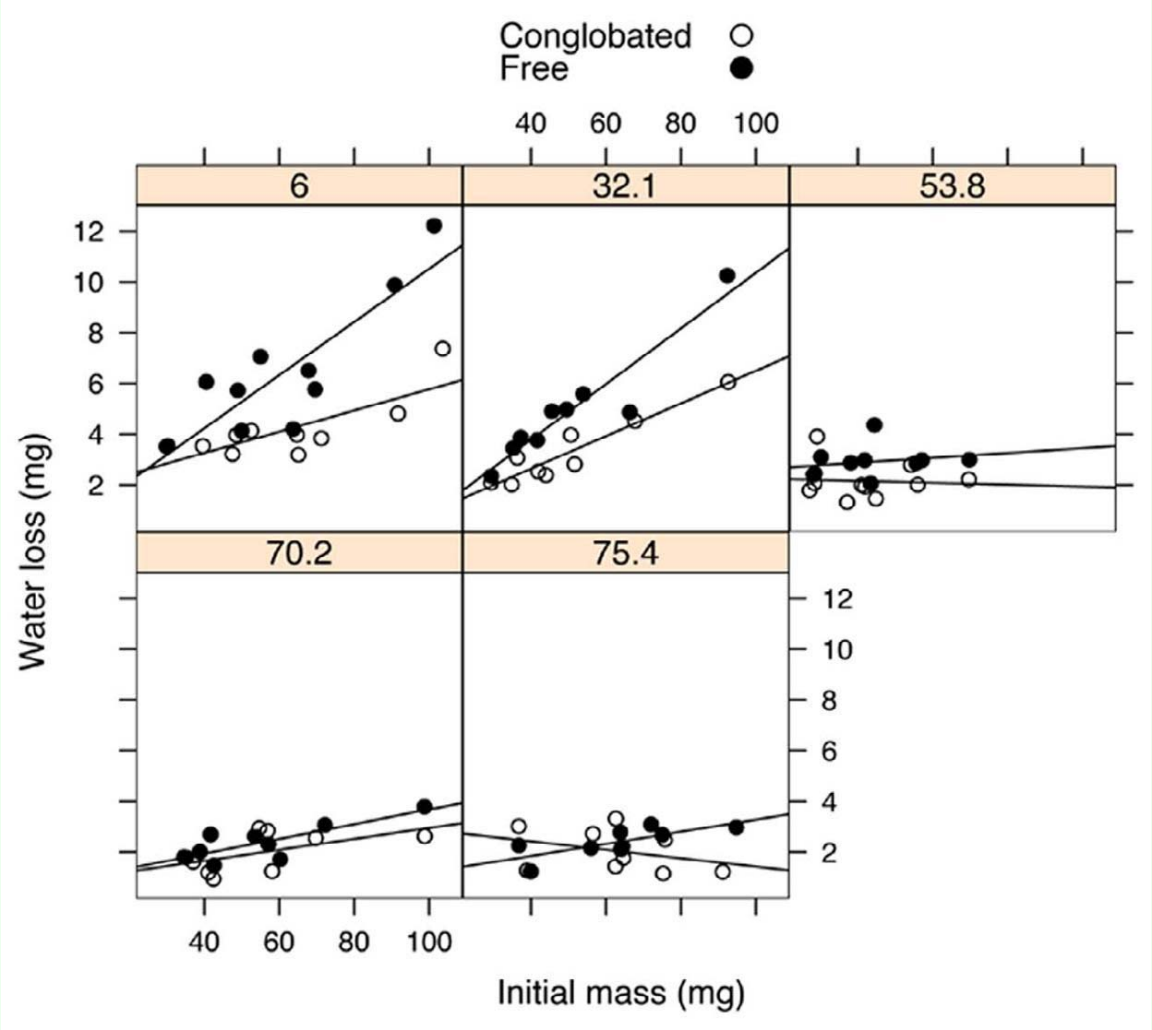
Water loss of *A. vulgare* in free and conglobated states, after 3 h in humidities ranging from 6.0 to 75.4 %RH.

At low humidities, A. vulgare lost more water when free than when conglobated, and water-loss rates increased with size (Figure 4). Water-loss rates declined as relative humidity increased. An ANCOVA was done for water loss at each humidity, with sex and conglobation state as factors and mass as a covariate. Conglobated isopods lost water less rapidly at 6–53 %RH (ANCOVA; P < 0.001 at 6 and 29 %RH; P < 0.04 at 53 %RH). A marginally significant effect of conglobation was detected at 70 %RH (ANCOVA; P = 0.054), but not at 75 %RH (P > 0.25).

## Discussion

Although the specimens of *A. vulgare* were obtained in the Mojave Desert in the southwestern United States, they were collected from a residential area that provided adequate moisture and protection from the elements that would probably be lacking in the open desert. *Armadillidium vulgare* is widespread today, but probably originated in the Mediterranean area (Souty-Grosset et al. 1998). Both microhabitat selection and local adaptation (Castaneda et al. 2004) may have helped *A. vulgare* to expand its range throughout the temperate regions of the world. On the other hand, Edney (1951) noted that the distribution of the more common isopods in the United Kingdom was by no means always related to moisture. For instance, *Armadillidium, Glomeris, Porcellio, Oniscus*, and *Philoscia* were all found in abundance in a single rubbish heap. *Armadillidium vulgare* was the only isopod present at the Mojave Desert collection site, but this may reflect the lack of other species in the area.

### Water-loss rates

*Armadillidium vulgare* lost water less rapidly when conglobated than when free (Figure 2). An important factor is the physical covering of the pleopods. Edney (1951, 1968) found that the pleopodal area was responsible for higher rates of water loss than the cuticle or other areas. Another factor is the reduced ratio of surface area to volume that occurs when isopods adopt a conglobated form. During conglobation, the volume of *A. vulgare* remains constant while the ventral surface is directed inward, greatly reducing the exposed surface area. In addition to being a source of higher water loss because of its respiratory function, the ventral surface also has a high surface area because it contains many long thin appendages and the tufts of invaginated tubules that form the “pseudotracheae” (Cloudsley-Thompson 1977).

Water-loss rates decreased as relative humidity increased (Figure 4). These findings were as expected, as the gradient for transpiration through the cuticle, as well as the gradient for evaporative water loss from respiratory surfaces, decreases at high humidities (Gibbs 1998, 2002). Above 53 %RH, water-loss rates did not differ significantly between conglobated and free isopods. It should be noted, however, that isopods still lost up to 5 % of their initial mass at 75 %RH, despite the short exposure period (3 hours). Although *A. vulgare* is a relatively xerictolerant isopod, it clearly is nowhere near as impermeable to transpiration as insects or most other terrestrial arthropods.

A potential problem in these experiments was boundary layer effects related to the still air conditions in the humidity vials. These would tend to reduce water-loss rates because of the reduced gradient for water loss at the surface of the isopod. However, water-loss rates at 6 %RH were comparable to those measured using flow-through respirometry. The calcium sulfate desiccant used in the respirometry measurements will dry air to <5 %RH, but not necessarily to 0 %RH (J.R.B. Lighton, Sable Systems International, personal communication). The concordance between data collected in flow-through conditions and in still air at 6 %RH indicates that boundary layer effects in the humidity vials did not substantially affect total water loss.

### Metabolic Rates

The rate of CO_2_ release was significantly reduced during conglobation. However, as noted in the results, one experimental group (males assayed free first, then conglobated) exhibited very low metabolism while conglobated, whereas metabolic rates in other groups declined by ∼20%. It is possible that CO_2_ accumulated in the tissues or in small air spaces inside conglobated isopods. Another possibility is anaerobic metabolism. These possibilities were not tested directly, but the modest decline in CO_2_ release suggests that conglobation does not completely block gas exchange, suggesting that anaerobic metabolism was unlikely.

Edney and Spencer (1955) found that blocking the pleopods did not affect release of CO_2_ from *A. vulgare*, suggesting free diffusion through the integument. In that study, however, experimental procedures could well have damaged the cuticle and increased permeability. Cuticular damage in our experiments is also a concern, but the consistent decrease in water-loss rates (in all 29 individuals) suggests that enforced conglobation did not cause substantial damage.

Given these considerations, the measurements of CO_2_ release should provide an accurate estimate of metabolic rates. Lower metabolic rates during conglobation may have been caused by the restriction of gross physical movement. In these experiments, the conglobated isopods appeared motionless, but it is unknown whether they were truly relaxed or were struggling to free themselves by flexing their muscles against the soft rubber band holding them closed. Four out of twenty individuals had higher metabolic rates when conglobated, and isometric muscle contractions may have contributed to these differences.

Non-conglobated isopods were often active, and we never observed spontaneous conglobation in either the respirometry chambers or in humidity vials. This is in contrast to Edney's (1951) observation that *A. vulgare* spontaneously conglobates in desiccating conditions. However, Edney's experiments were performed at temperatures above 40°C, conditions that *A. vulgare* avoids in nature. Klok et al. (2004) instead noted increased activity in *A. vulgare* above 40°C, as indicated by activity meters (C. J. Klok, Arizona State University, Tempe, Arizona USA, personal communication), but those experiments were performed at relatively high humidities. Perhaps the combination of high temperature and low humidity used by Edney (1951) stimulated conglobation behavior.

Alternatively, handling stress could well have induced conglobation and reduced metabolic rate anyway. Parson and Hoffmann (1993) have proposed that lower metabolic rates are a common response to environmental stress. Reduced metabolism during conglobation would presumably have adaptive value for individuals trying to survive desiccating conditions of unpredictable duration, because it would allow individuals to remain conglobated longer.

### Summary

Water loss via the respiratory surface is one of the most important physiological factors affecting the survival and distribution of terrestrial isopods (Edney 1968; Cloudsley-Thompson 1969, as cited in Nair et al. 2003). Our flow-through respirometry data support the hypothesis that conglobation behavior of *A. vulgare* reduces water loss at low humidities. The data from the trials at known humidity suggest that conglobation also decreases water loss at sub-saturating humidity. Further research should focus on the ability of conglobation to actually limit the flow of air passing over the pleopods and relate that to changes in the rate of water loss. In addition, experiments that examine water-loss measurements during spontaneous, rather than forced, conglobation are needed.
